# Viscoelastic blood coagulation testing system enabled by a non‐contact triboelectric angle sensor

**DOI:** 10.1002/EXP.20230073

**Published:** 2023-11-23

**Authors:** Baocheng Wang, Xuelian Wei, Hanlin Zhou, Xiaole Cao, Enyang Zhang, Zhong Lin Wang, Zhiyi Wu

**Affiliations:** ^1^ Beijing Institute of Nanoenergy and Nanosystems Chinese Academy of Sciences Beijing China; ^2^ School of Nanoscience and Technology University of Chinese Academy of Sciences Beijing China; ^3^ Georgia Institute of Technology Atlanta Georgia USA

**Keywords:** angle sensor, blood coagulation testing, non‐contact, thromboelastography, triboelectric nanogenerator

## Abstract

Thromboelastography (TEG) remains a convenient and effective viscoelastic blood coagulation testing device for guiding blood component transfusion and assessing the risk of thrombosis. Here, a TEG enabled by a non‐contact triboelectric angle sensor (NTAS) with a small size (∼7 cm^3^) is developed for assessing the blood coagulation system. With the assistance of a superelastic torsion wire structure, the NTAS‐TEG realizes the detection of blood viscoelasticity. Benefiting from a grating and convex design, the NTAS holds a collection of compelling features, including accurate detection of rotation angles from −2.5° to 2.5°, high linearity (*R* ^2^ = 0.999), and a resolution of 0.01°. Besides, the NTAS exhibits merits of low cost and simplified fabrication. Based on the NTAS‐TEG, a viscoelastic blood coagulation detection and analysis system is successfully constructed, which can provide a graph and parameters associated with clot initiation, formation, and stability for clinicians by using 0.36 mL of whole blood. The system not only validates the feasibility of the triboelectric coagulation testing sensor, but also further expands the application of triboelectric sensors in healthcare.

## Graphical abstract

Thromboelastography (TEG) remains a convenient and effective viscoelastic coagulation testing device. This paper presents a novel TEG enabled by a miniaturized triboelectric coagulation sensor, which can realize a comprehensive assessment of the coagulation system and is expected to promote the miniaturization of traditional TEGs.

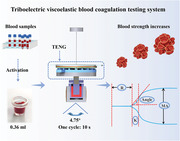

## 1 INTRODUCTION

The process of blood coagulation is a series of enzyme‐linked reactions that occur when coagulation factors are activated by endogenous or exogenous activators.^[^ ^1^ ^]^ Blood coagulation testing can assess the blood coagulation process, which is of great significance for the diagnosis of coagulation‐related diseases, screening of anticoagulant drugs, and evaluation of coagulation status during the perioperative period.^[^ ^2^ ^]^ However, routine coagulation tests such as prothrombin time (PT), thrombin time (TT), and activated partial thromboplastin time (APTT) are severely limited by long waiting time for results (45–60 min) and scarce information for platelet function.^[^ ^3^ ^]^

Thromboelastography (TEG)^[^ ^4^ ^]^ is a point‐of‐care viscoelastic coagulation testing device that overcomes the above limitations and provides a graph reflecting the full picture of coagulation in 30 min using less than 1 mL of whole blood. Besides, the testing temperature of the TEG can be adjusted according to the patient's temperature. During coagulation cascade,^[^ ^5^ ^]^ fibrin, platelets, and blood cells form an insoluble three‐dimensional (3D) cross‐linked clot. The viscoelasticity of the clot varies with time prolonging. The principle of the TEG is to detect the viscoelasticity of a blood sample under a low shear condition.^[^ ^6^ ^]^ The core sensing element of the TEG is an electromagnetic angle sensor, commonly consisting of a permanent magnet and coils, which can transform blood viscoelasticity into electrical signals. Thus, a graph related to blood viscoelasticity can be acquired after a test. Nevertheless, the large size of the electromagnetic angle sensor limits the miniaturization of the TEG and is not consistent with the development of portable medical test devices.

Based on the coupling of contact electrification and electrostatic induction, triboelectric nanogenerators (TENGs) are burgeoning with the merits of light weight, easy miniaturization, outstanding low‐frequency motion monitoring, multiple structures, and low cost.^[^ ^7^ ^]^ As self‐powered sensors, TENGs are widely applied in healthcare monitoring,^[^ ^8^ ^]^ human–machine interfacing,^[^ ^9^ ^]^ and robotic sensing.^[^ ^10^ ^]^ Meanwhile, triboelectric angle sensors based on TENGs have been demonstrated in a wide range of angle sensing applications, ranging from joint bending angle sensing,^[^ ^11^ ^]^ steering wheel rotation angle sensing^[^ ^12^ ^]^ to tilt angle sensing.^[^ ^13^ ^]^ Among them, the triboelectric rotation angle sensor is mainly composed of a rotor that follows the motion of the sensing object and a stator that detects the motion of the rotor. The rotor is usually in close contact with the stator to improve the performance of the sensor, but this also increases the resistance between the rotor and the stator. In the TEG, the force that drives the rotor is initiated from blood coagulation. The force is small, which is not sufficient to overcome the resistance between the rotor and the stator. Non‐contact mode triboelectric sensor can effectively minimize the resistance. Therefore, a miniaturized triboelectric angle sensor in non‐contact mode is highly desired for the further miniaturization of the TEG.

In this work, we proposed a non‐contact triboelectric angle sensor (NTAS) as the core sensing element of the TEG for the analysis of coagulation‐related diseases. The NTAS retains a small size of about 7 cm^3^, which is expected to facilitate the miniaturization of the TEG. By leveraging a torsion wire structure, the NTAS‐TEG assesses clot viscoelasticity by detecting the rotation angle of the probe, which is verified through finite element simulation of the NTAS‐TEG. Mainly fabricated by a printed‐circuit‐board technique and a 3D‐printed technique, the NTAS is a grating structured TENG, exhibiting merits of low cost and simplified fabrication. Benefiting from an elaborate design, rotation angles ranging from −2.5° to 2.5° can be accurately identified with a decent linearity, a sensitivity of 0.58 V deg^−1^, and a resolution of 0.01°. The performance of the NTAS fluctuates at an average of 3.5% over 20,000 cycles, exhibiting great durability. Finally, a blood coagulation detection and analysis system for the NTAS‐TEG was successfully constructed, which can provide a graph and parameters (reaction time [R], kinetic time of clot formation [K], clot formation rate [Angle], and maximum amplitude [MA]) related to the function of the blood coagulation system for clinicians.

## 2 RESULTS AND DISCUSSION

### 2.1 Design of the NTAS‐based TEG

**FIGURE 1:**
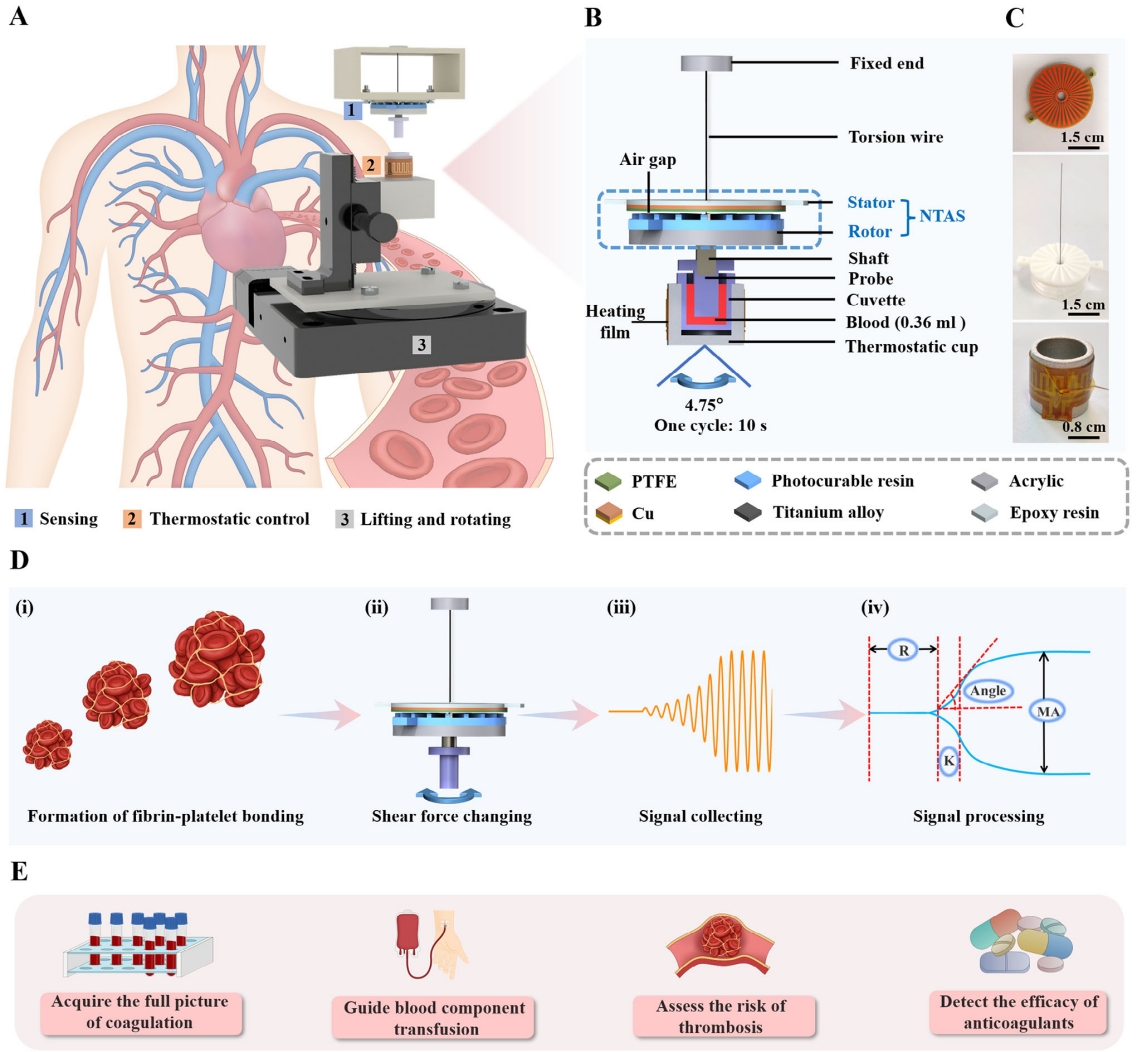
Design of the non‐contact triboelectric angle sensor (NTAS)‐based thromboelastography (TEG). A) Schematic diagram of the NTAS‐TEG. B) Simplified diagram of the NTAS‐TEG with sectional view of the cuvette during a test. C) Photographs of the interdigital electrodes board, the NTAS's rotor connected to a torsion wire, and the thermostatic cup covered with a flexible heating film and a small temperature sensor. D) Working mechanism of the NTAS‐TEG‐based blood coagulation testing and analysis system consisting of four steps ((i)–(iv)). E) Potential application scenarios of the NTAS‐TEG.

We proposed a TEG based on the NTAS for detecting coagulation‐related diseases (Figure 1A and Figure S1A). The NTAS‐TEG consists of a sensing module, a thermostatic control module, and a lifting and rotating module, allowing the blood coagulation system to be evaluated with only 0.36 mL of whole blood. Photographs of different modules of the NTAS‐TEG are shown in Figure S1B–E. To illustrate the NTAS‐TEG clearly, a simplified diagram of the NTAS‐TEG during the testing of a blood sample is presented in Figure 1B. The sensing module is mainly composed of the NTAS, a superelastic torsion wire structure (diameter, 0.2 mm), and a probe. The sensing module converts the clot strength into the torsion angle of the torsion wire, which can be detected by the NTAS, thereby transforming the clot strength to the electrical signals. The thermostatic control module consists of a thermostatic cup covered with a flexible heating film and a small temperature sensor, and a control unit, providing an environment of constant temperature for the blood sample (Figure 1C). The rotating module offers a low shear field for the test of blood viscoelasticity. The thermostatic cup can move down or up under the control of the lifting module. The simplified testing process of the NTAS‐TEG is as follows. During a test, the blood is incubated in a cylindrical cuvette at the temperature of the patients. The probe suspended by the torsion wire is placed in the cuvette. The cuvette oscillates through an angle of 4°45′ relative to the probe with a period of 10 s (the rotation process of the rotating module in each cycle is shown in Table S1). A graph related to blood viscoelasticity can be obtained within about 30 min.

As the core sensing element of the TEG, the NTAS is a grating structured TENG composed of a rotor and a stator, holding the advantages of small size (diameter, 30 mm; height, 10 mm), high accuracy, simple fabrication, and low cost. There is an air gap of 100 μm between the rotor and the stator, which minimizes the resistance between them. The stator includes a group of interdigital copper electrodes, an epoxy resin base, and a polytetrafluoroethylene (PTFE) film with high electron affinity pasted on the interdigital electrodes. The rotor includes a layer of 3D‐printed photocurable resin with a grating convex structure and acrylic bases. The grating and convex structure endows the NTAS with large outputs in the non‐contact mode. The stator is installed on the bottom of a 3D‐printed plastic box with a cavity, while the rotor is suspended by a torsion wire fixed to the top of the box and passes coaxially through the stator, tightly connected to the probe by a shaft. The rotor rotates along with the probe during testing a blood sample. Photographs of the interdigital electrodes board and the rotor connected to the torsion wire are shown in Figure 1C. All parts of the sensing module are coaxially assembled, the specific dimensions and the schematic diagram of the assembling process are illustrated in Figures S2 and S3, respectively. The detailed assembling process is described in the Experimental Section.

The working principle of the NTAS‐TEG is illustrated in Figure 1D(i)–(iv). At first, the movement of the cuvette does not affect the probe. As the fibrin–platelet bonding forms between the probe and the cuvette, the rotation of the cuvette is transmitted to the probe. Due to the change of the blood viscoelasticity, the shear force applied to the probe by the blood changes, resulting in the change of the rotation angle of the probe. Therefore, an oscillating waveform signal can be generated by the NTAS. Finally, a trace and several key parameters (R, K, Angle, MA) are extracted through the acquired signal. In short, the NTAS‐TEG assesses the blood coagulation function by detecting the rotation angle of the probe, demonstrating its potential in acquiring the full picture of coagulation, guiding blood component transfusion, assessing the risk of thrombosis, and detecting the efficacy of anticoagulants (Figure 1E).

### 2.2 Mechanical and electrostatic simulation of the NTAS‐based TEG

**FIGURE 2:**
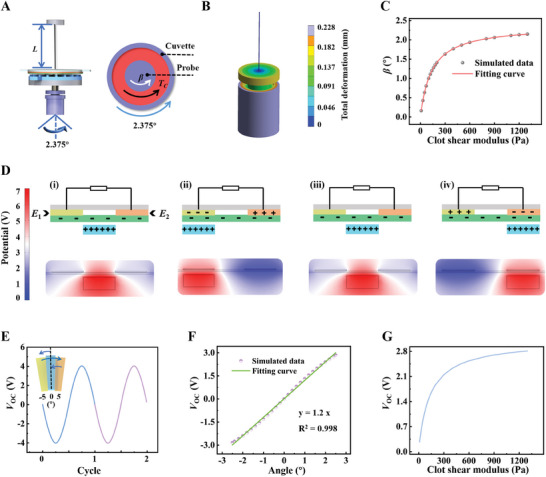
Mechanical and electrostatic simulation of the non‐contact triboelectric angle sensor (NTAS)‐based thromboelastography (TEG). A) Mechanical analysis and B) total deformation simulation of the simplified NTAS‐TEG when the rotation angle of the cuvette is 2.375° and the clot shear modulus is 400 Pa. C) *β* at different clot shear modulus from 10 to 1300 Pa. D) Bidirectional rotation angle sensing mechanism of the NTAS and corresponding electric potential simulation. E1 and E2 represent the left electrode and the right electrode, respectively. E) Simulation of *V*_OC_ waveforms when the rotor rotates from −5° to 5°. F) Simulation of *V*_OC_ amplitude when the rotor rotates from −2.5° to 2.5°. G) Relationship between *V*_OC_ and the clot shear modulus.

To demonstrate the feasibility of the NTAS‐TEG, the mechanical and electrostatic simulations of the NTAS‐TEG were conducted through a finite element simulation software. To analyze the static field clearly, two assumptions are set as follows: (1) the annulus clot does not separate from the inner wall of the cuvette and the outer wall of the probe; (2) the clot is regarded as a low elastomer, thus the viscosity of the clot is neglected. Static analysis of the NTAS‐TEG when the rotation angle of the cuvette is 2.375° as shown in Figure 2A. A shear deformation of the clot is generated under the shear force of the cuvette, and the shear force is transmitted to the probe, namely the torque *T*_c_ in Figure 2A. Driven by the *T*_c_, the probe rotates through an angle of *β*. The *T*_c_ can balance with the torque generated by the torsion wire, which can be expressed as the following formula:

(1) $$2.375^{\circ }>\beta =\frac{T_{c}\cdot L}{I_{p}\cdot G_{w}\;}$$

where *β* represents the rotation angle of the probe, *T*_c_ the torque applied to the probe by the clot, *L* the length of the torsion wire, *I*_p_ the polar moment of inertia of the torsion wire, and *G*_w_ the shear modulus of the torsion wire. Considering that *L, I*_p_, and *G*_w_ are the structural parameters of the NTAS‐based sensing module, the *β* mainly depends on the *T*_c_ *_,_* which correlates with the clot strength. As illustrated in Figure 2B, the total deformation was simulated when the clot shear modulus was set as 400 Pa.^[^ ^14^ ^]^ And the other simulation parameters are shown in Table S2. The total deformation becomes larger as the radius of the clot becomes larger because the deformation is rotational. The largest deformation occurs at the outermost edge of the cuvette. As shown in Figure 2C, the simulation of the *β* at different clot shear modulus is acquired and the fitting curve is obtained by nonlinear fitting. Notably, the *β* has a specific relationship with the clot shear modulus_._ As a result, it is viable to characterize the clot shear modulus by measuring the *β*. The rotor of the NTAS rotates together with the probe, which can be detected by the stator of the NTAS.

Corresponding to the rotation process of the rotating module every cycle, the bidirectional rotation angle sensing mechanism of the NTAS is illustrated in Figure 2D. The left and the right electrodes are designated as *E*_1_ and *E*_2,_ respectively. And *V*_2_ − *V*_1_ is set as the open‐circuit voltage (*V*_OC_) of the NTAS. The corresponding simulation parameters are shown in Table S3. In the initial state, the rotor grid is located at the center of adjacent stator grids and there is an air gap between the rotor and the stator. The surfaces of the rotor and the PTFE are charged with opposite and equivalent charges based on the contact electrification of the air. The PTFE is electropositive because of its high electron affinity. There is no electric potential difference between *E*_1_ and *E*_2_ based on the electrostatic induction (Figure 2D(i)). Then, as the rotor moves to the left, the positive charges are driven from *E*_1_ to *E*_2_ under the electric potential difference, and *V*_OC_ gradually decreases. *V*_OC_ achieves the minimum as the rotor is completely coincident with *E*_1_ (Figure 2D(ii)). Next, as the rotor moves back to the initial state, the positive charges flow back from *E*_2_ to *E*_1_, thus *V*_OC_ gradually returns to zero as the rotor returns to the initial state (Figure 2D(iii)). As the rotor continues to move to the right, *V*_OC_ achieves the maximum when the rotor is completely coincident with *E*_2_ (Figure 2D(iv)). *V*_OC_ decreases to zero as the rotor returns to the initial state (Figure 2D(i)). The corresponding simulated electric potential diagrams are shown at the bottom of Figure 2D. More simulations can be found in Figure S4. Consequently, *V*_OC_ changes corresponding with the cycle of the rotating module are clarified clearly. The corresponding *V*_OC_ waveforms of two cycles are shown in Figure 2E. *V*_OC_ exhibits excellent linearity with the rotation angles from −2.5° to 2.5° (Figure 2F). Combining the simulation of Figure 2C,F, a relationship curve between the clot shear modulus and *V*_OC_ is acquired, indicating that *V*_OC_ can be used to characterize the clot elasticity (Figure 2G). The simulation above successfully clarifies the mechanism and demonstrates the feasibility of the NTAS‐TEG.

### 2.3 Optimization, sensing performance, and stability of the NTAS

**FIGURE 3:**
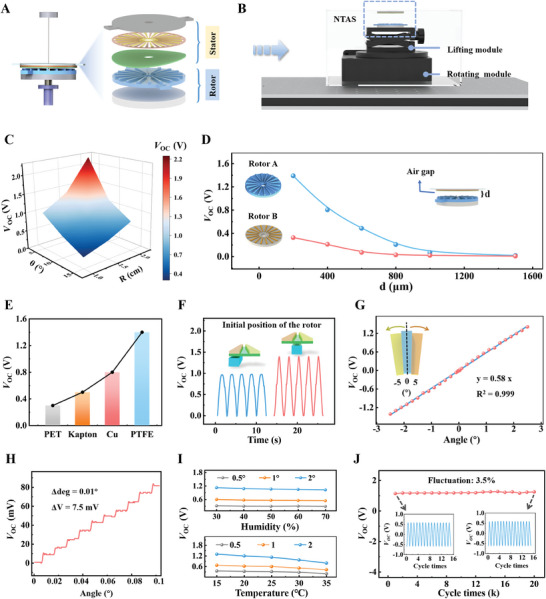
Optimization, sensing performance, and stability of the non‐contact triboelectric angle sensor (NTAS). A) Exploded diagram of the NTAS. B) Measurement platform of the NTAS. C) *V*_OC_ at the *θ* of 5°, 10°, and 15° when the radius is 1, 1.5, and 2 cm, respectively. D) *V*_OC_ of Rotor A and Rotor B with *d* ranging from 100 to 1500 μm. E) *V*_OC_ of different stator triboelectric layers (polyethylene terephthalate, Kapton, Cu, and polytetrafluoroethylene). F) *V*_OC_ of different initial relative positions of the stator and rotor. G) *V*_OC_ with rotation angles from −2.5° to 2.5°. H) *V*_OC_ with a rate of 0.01° per step from 0° to 0.1°. I) *V*_*OC*_ at different environmental temperatures and humidity. J) Durability test results of the NTAS.

The NTAS is the core sensing element of the TEG, comprising a rotor and a stator, as illustrated in Figure 3A. A non‐contact structure is adopted to lessen the friction force. A grating structured TENG is leveraged to fabricate the NTAS, which can effectively magnify the outputs at a settled small rotation angle. A measurement platform of the NTAS is illustrated in Figure 3B. The rotation angle of the rotor and the distance of the air gap (*d*) between the rotor and stator are adjusted by a rotating module and a lifting module, respectively. Note that the rotation angle of the rotor is set as 2.375° in the following measurements. The *θ* (central angle of interdigital electrodes and rotor's convex grids) and the *R* (radius of the NTAS) are the main factors influencing the outputs, since they determine the magnitude of the induction area once the rotation angle is settled. *V*_OC_ elevates with increasing *R* and decreasing *θ* due to the amplification of the induction area (Figure 3C). Considering the miniaturization and larger outputs of the NTAS, a *θ* of 5° and an *R* of 1.5 cm are determined for the NTAS. Furthermore, the effects of different rotors and the *d* on the outputs of the NTAS are discussed (Figure 3D). Rotor A is made of 3D‐printed photocurable resin with grids thickness of 1 mm and Rotor B is fabricated through printed‐circuit‐board technique (thickness of copper, 35 μm). Obviously, *V*_OC_ of Rotor A is greater than that of Rotor B when the *d* is the same. Besides, the corrosion resistance of photocurable resin is superior to that of copper, endowing the NTAS greater stability. Therefore, Rotor A is a better candidate for the rotor of the NTAS. *V*_OC_ enhances as the *d* decreases from 1500 to 100 μm. When the *d* is further reduced, it is difficult to integrate the NTAS. Hence the *d* of 100 μm is settled for the NTAS. In addition, reasonable material selection of the triboelectric layer for the stator is crucial for the performance of the NTAS. Polyethylene terephthalate (PET), Cu, Kapton, and PTFE are set as the candidates, which are the commonly used triboelectric materials with high charge density. Among them, Cu and PET are triboelectric positive materials, while PTFE and Kapton are triboelectric negative materials. Moreover, Cu is a metal, while PTFE, Kapton, and PET are polymer materials. These considerations ensure the rationality of material selection. Compared to the PET, Cu, and Kapton, *V*_OC_ of the PTFE is the largest because of the strong electron affinity of the PTFE^[^ ^15^ ^]^ (Figure 3E). As a result, the PTFE is chosen as the triboelectric layer of the stator. To ensure that the NTAS can measure bidirectional rotation angles, the initial position of the rotor grids completely coincides with grating electrodes or intervals between adjacent electrodes. It can be observed that *V*_OC_ of the right condition is larger than that of the left condition (insets of Figure 3F). Accordingly, it is a better choice to set the initial position of the rotor grids at the intervals of adjacent stator electrodes.

To investigate the rotation angle sensing characteristic of the NTAS, *V*_OC_ of rotation angles from −2.5° to 2.5° was measured and a fitting curve was obtained (Figure 3G). *V*_OC_ waveforms of 0.01°, 0.02°, 1.5°, and 2.3° are illustrated in Figure S5A,B. The square of the correlation coefficient is 0.999, which represents a linear error of less than 0.1%, confirming the simulation results of Figure 2F. The sensitivity of the NTAS is defined as the following equation:

(2) $$S=\frac{▵V}{▵A}$$

where *S* represents the sensitivity of the NTAS, and Δ*V* is the voltage variance corresponding to Δ*A* that is the rotation angle variance. A sensitivity of 0.58 V deg^−1^ of the NTAS can be achieved. Every step rotation of 0.01° around 0° could be evidently distinguished with an average voltage variance of 75 mV, indicating that the resolution of the NTAS can reach 0.01° (Figure 3H). It is noteworthy that the sensitivity and resolution can be further improved by optimizing the material selection and structural design of the NTAS. As the maximum absolute value of the probe rotation angle that is less than 2.375° is within the detection range of the NTAS, the NTAS can meet the requirements of the TEG. To examine the humidity and temperature resistance of the NTAS, *V*_OC_ of 0.5°, 1°, and 2° at different humidities (30%−70%) and temperatures (15–35°C) were measured, respectively, as illustrated in Figure 3I. *V*_OC_ waveforms of 1° at different humidities and temperatures are illustrated in Figure S5C,D. *V*_OC_ experiences a slight drop with increasing humidity, while *V*_OC_ experiences a larger reduction with increasing temperature. As the NTAS‐TEG is used indoors, the influence of humidity and temperature can be minimized to some extent. In future works, the humidity and temperature resistance can be further modified by encapsulating the NTAS. The fatigue test results of the NTAS are illustrated in Figure 3J (the rotor rotates between −1° and 1° with 20,000 cycles). Benefiting from the non‐contact structure of the NTAS, *V*_OC_ fluctuates at an average of 3.5%, indicating excellent durability. The NTAS with the advantages of high accuracy, simple fabrication, low cost, and great durability is successfully demonstrated by the above experimental results, which is competent to sense the small rotation angle of the NTAS‐TEG. Besides, the NTAS retains a small size of ∼7 cm^3^, which is expected to facilitate the miniaturization of the TEG.

### 2.4 Blood sample tests and a signal analysis method of the NTAS‐based TEG

**FIGURE 4:**
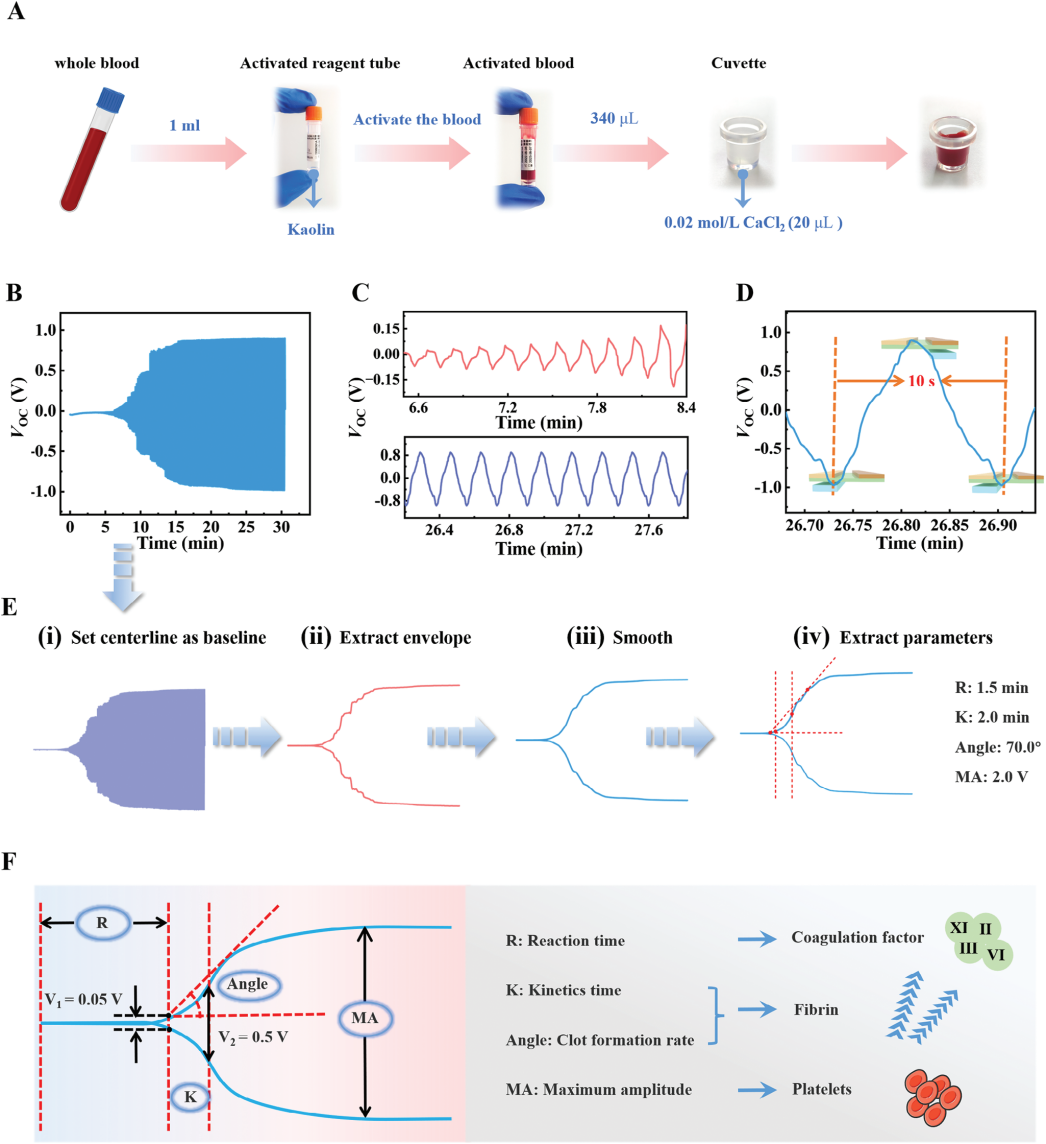
Blood sample tests and a signal analysis method of the non‐contact triboelectric angle sensor (NTAS)‐based thromboelastography. A) Flowchart of the activation process of a blood sample. B) *V*_OC_ waveform of a porcine blood sample. C) Details of the *V*_OC_ waveform of B). D) *V*_OC_ waveform of one cycle. The insets show the corresponding NTAS motion status. E) Signal analysis process from the original *V*_OC_ waveform to the final trace. F) Rules of extracting R, K, Angle, and MA, and the corresponding relationship between the parameters and blood cells.

The testing of real blood samples is critical to validate the function of the NTAS‐enabled TEG. Considering that the porcine blood coagulation system is similar to the human blood coagulation system, standardized citrated porcine whole blood was used to investigate the performance of the NTAS‐TEG. Detailed measuring steps of the NTAS‐TEG are shown in the Experimental Section and Movie S1, among which the activation process of the blood sample before the test is shown in Figure 4A. A *V*_OC_ waveform was obtained by measuring a porcine whole blood sample, which is similar to the graph of commercial TEG^6^ (Figure 4B). The first 10 s of the test results are discarded to eliminate the initial interference of the measuring device and operators, the test results of the first 10 s are discarded. Initially, the blood is in the activation period where the blood has not started coagulating yet and the corresponding *V*_OC_ waveform is a straight line. After the blood experiences through coagulation cascade, fibrinogen is transformed into fibrin under the catalysis of thrombin, then insoluble clots begin to form. The *V*_OC_ amplitude is small because a sparse fibrin network results in a weak clot strength. With the further progress of blood coagulation, the fibrin network becomes denser, and platelets further gather. The clot strength gradually enhances, and *V*_OC_ amplitude further enlarges. About 20 min later, the clot strength reached the maximum, and the corresponding *V*_OC_ amplitude also reached the maximum. The corresponding *V*_OC_ waveforms are shown in Figure 4C when the clot strength is at the growth and the stable period. In addition, the motion process of the NTAS and the corresponding *V*_OC_ waveforms are shown in Movie S2. Notably, the corresponding *V*_OC_ amplitude gradually enlarges with the increasing rotation angle of the rotor. Moreover, a *V*_OC_ waveform of one cycle is investigated in Figure 4D. It can be observed that the time interval between two adjacent valleys is 10 s, corresponding to one cycle of the rotating platform. The rotor moves to the maximum negative angle of one cycle when *V*_OC_ reaches the valley value, while the rotor moves to the maximum positive angle of one cycle when *V*_OC_ reaches the peak value.

To extract a trace and parameters related to the function of the blood coagulation system from the signals of blood samples, a signal analysis method was developed for the NTAS‐TEG (Figure 4E). The process is as follows: (i) set the centerline of the *V*_OC_ waveform as the baseline, then subtract the baseline from the original waveform; (ii) extract the envelope of the waveform and ensure that the envelope fits the peak and valley points; (iii) smooth the envelope to obtain the final trace; (iv) extract the parameters according to the extraction rules of Figure 4F. The extraction rules are as follows: R equates to the time taken from the start to the point where the trace amplitude reaches 0.05 V; K equates to the time taken from R point to the point where the amplitude reaches 0.5 V; Angle equates to the angle between the horizontal line and the tangent of the trace through the R point (the amplitude is expanded by 20 times when calculating the angle to ensure a better match between the angle and the image effect); MA equates to the maximum amplitude. The parameters represent different periods of blood coagulation. Among these parameters, R represents a period from the beginning of blood coagulation to initial fibrin formation; K represents the time taken to reach a certain degree of clot strength; Angle represents the rate of fibrin polymerization and cross‐linking; MA represents the maximum clot strength. As a result, the extracted parameters can assess the function of different blood components (Figure 4F). After determining the normal range of each parameter, the parameters can be utilized to detect the relevant diseases. For example, if R is prolonged, the blood sample may contain anticoagulants or lack of coagulation factors, indicating that the patient is at risk of bleeding. And the enlargement of MA usually means the patient is at risk of thrombosis.

### 2.5 Viscoelastic blood coagulation detection and analysis system enabled by the NTAS‐based TEG

**FIGURE 5:**
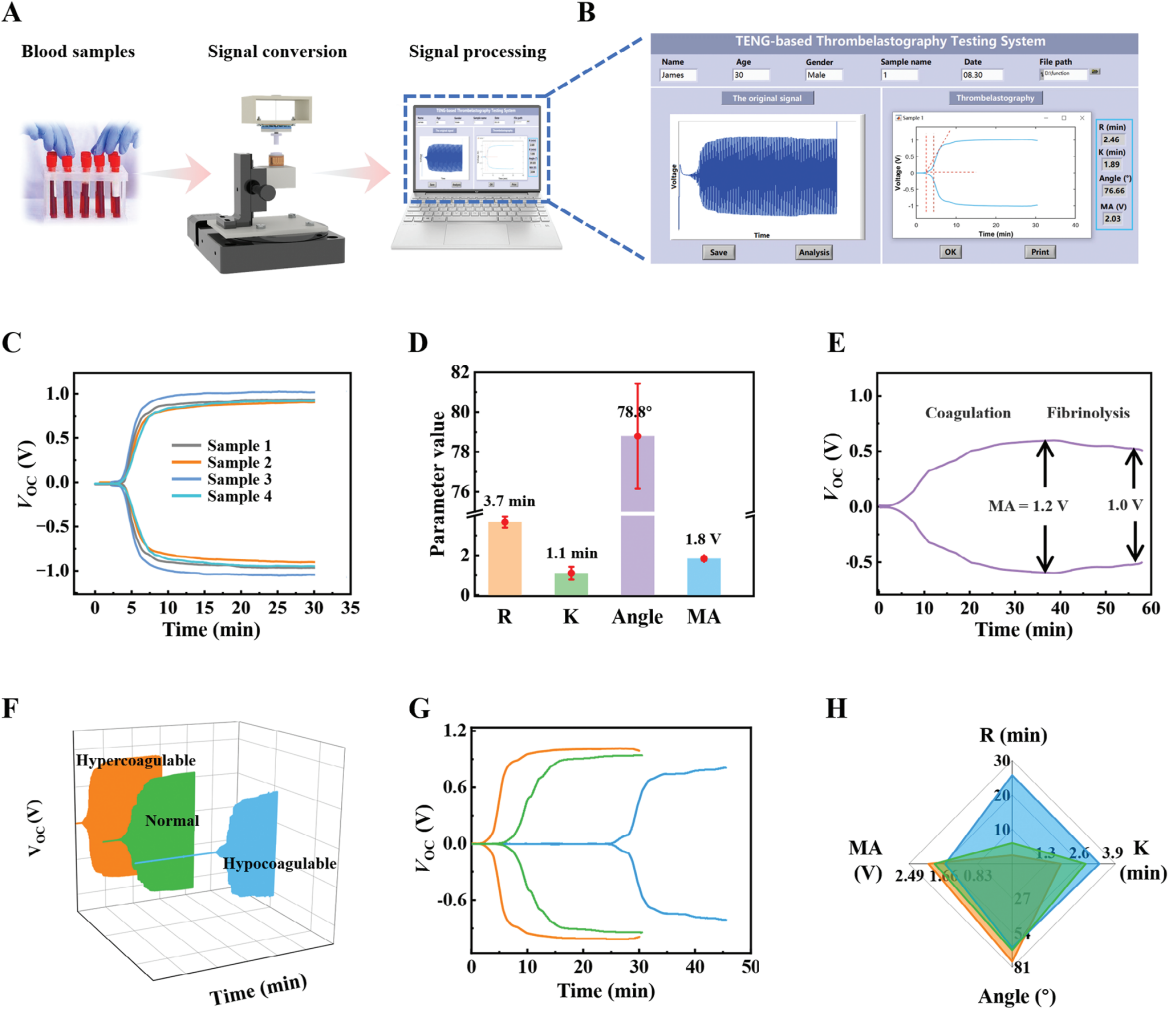
Viscoelastic blood coagulation detection and analysis system with the non‐contact triboelectric angle sensor‐based thromboelastography. A) Working process of the system. B) LabVIEW front panel of the system when testing a blood sample. C) Voltage traces of four porcine blood samples from the same batch. D) Error diagram of parameters (R, K, Angle, and MA) extracted from the voltage traces of four blood samples. E) Voltage traces of a porcine blood sample with extended detection time. F) Original data, G) traces, and H) parameters (R, K, Angle, and MA) of hypocoagulable (blue), normal (green), and hypercoagulable (orange) quality control samples.

To realize testing the blood coagulation and signal analysis, a viscoelastic blood coagulation detection and analysis system based on the signal analysis method of Figure 4 was successfully constructed consisting of the NTAS‐TEG, an electrometer, a data acquisition card, and a laptop (Figure S6). The working process of the signal test system is illustrated in Figure 5A. Then 0.36 mL of a blood sample is activated and added into the cuvette. The NTAS transforms the clot strength of the blood sample into voltage signals, which can be measured by the electrometer, transmitted to the laptop via the data acquisition card, and processed by a program based on the LabVIEW software. The original data, the voltage trace, and the extracted parameters are displayed on the screen of the laptop (Figure 5B). To verify the repeatability of the NTAS‐TEG, four blood samples pipetted from the same batch of citrated porcine whole blood samples were tested by the NTAS‐TEG. The voltage traces of the four samples are illustrated in Figure 5C. The error diagram of parameters (R, K, Angle, and MA) extracted from the four traces is shown in Figure 5D. It can be observed that the repeatability of these four traces is great to some extent. The fibrinolysis process of blood can be detected by prolonging the test time. As shown in Figure 5E, the *V*_OC_ amplitude of a citrated porcine whole blood sample reaches the maximum (1.2 V) at 37.5 min and declined by 16.7% (1.0 V) at 58 min. It can be inferred that the clot strength reaches the maximum at 37.5 min and then declined on account of fibrinolysis. This proves that the NTAS‐TEG can detect the coagulation and fibrinolysis processes simultaneously by extending the detection time. Detection of both blood coagulation and fibrinolysis can provide a more comprehensively assessment of the blood coagulation system. In this work, we aim to preliminarily verify the performances of the NTAS‐TEG, so that only the coagulation process is detected in most tests. To further demonstrate the accuracy of the NTAS‐TEG, hypocoagulable, normal, and hypercoagulable quality control samples were tested by the system. The corresponding original *V*_OC_ waveforms, traces, and coagulation parameters are illustrated in Figures 5F and 5G, respectively. The accurate parameter values are illustrated in Table S4. Figure 5H shows the differences in coagulation parameters among different samples. In the order of hypocoagulable, normal, and hypercoagulable samples, R and K decrease, while Angle and MA increase. This is consistent with the characteristics of the hypocoagulable, normal, and hypercoagulable samples. The dynamic voltage waveforms of hypocoagulable and hypercoagulable samples during the tests are shown in Movies S3 and S4. These test results demonstrate the repeatability and accuracy of the NTAS‐TEG and validate the feasibility of the triboelectric coagulation detection sensor.

## 3 CONCLUSION

In summary, a TEG enabled by the NTAS was constructed for comprehensively assessing the blood coagulation system. The NTAS retains a small size of about 7 cm^3^, which is expected to facilitate the miniaturization of the TEG. Through a torsion wire and a probe, the clot deformation is transformed into the rotation of the probe, which can be detected by the NTAS. Thus, the output voltage of the NTAS can be used to characterize the clot strength, which is verified by the finite element simulation of the NTAS‐TEG. The 3D‐printed technique and printed‐circuit‐board technique are adopted to fabricate the NTAS, endowing the NTAS with the merits of low cost and easy fabrication. The NTAS can accurately identify the rotation angle from −2.5° to 2.5° with a high linearity, a sensitivity of 0.58 V deg^−1^, and a resolution of 0.01°. Benefiting from the non‐contact mode, the NTAS realizes great durability with the output voltage fluctuating at an average of 3.5% over 20,000 cycles. With the assistance of a signal analysis method, a viscoelastic blood coagulation detection and analysis system based on the NTAS‐TEG can provide a trace and parameters associated with blood coagulation by using 0.36 mL of whole blood. The system realizes effective analysis of a hypocoagulable quality control sample and a hypercoagulable quality control sample, validating the triboelectric coagulation testing sensor and expanding the application of triboelectric sensors for healthcare.

## 4 EXPERIMENTAL SECTION

### 4.1 Materials

Citrated porcine whole blood (sodium citrate, 0.109 mol L^−1^, HONGQUAN BIO). Materials purchased from UD‐Bio: cuvette (plastic), probe (plastic), CaCl_2_ solution (0.2 mol L^−1^), activation reagent tube (activation reagent, Kaolin), hypocoagulable, normal, and hypercoagulable quality controls (lyophilized citrated animal plasma and buffer solution).

### 4.2 Fabrication of the sensing module based on the NTAS

Sheet 1, sheet 2, sheet 3, and sheet 4 were fabricated by cutting a 2 mm thickness acrylic sheet by a laser cutting machine. A box with a cavity and the functional layer of the rotor (grids, thickness, 1 mm; base, thickness, 1 mm) were fabricated by a photocurable 3D printing machine. The interdigital electrodes board (copper, thickness, 35 μm; epoxy resin, thickness, 1.6 mm) was fabricated through printed‐circuit‐board technique. The specific dimensions of the components above are illustrated in Figure S2. The fabrication process of the sensing module can be divided into four steps, as illustrated in Figure S3. (A) Fabrication of the NTAS's rotor integrated with a torsion wire of 25 mm length and a shaft of 6 mm length. (B) Fabrication of the NTAS's stator pasted with a 50 μm thickness PTFE film. (C) Set the relative position of the rotor and the stator: The rotor was fixed on the box with screw bolts. And a PTFE film with thickness of 100 μm was clamped between the rotor and the stator to determine the thickness of the gap. The design of the box holes (Figure S2D) can make the rotor grids locate at the center of the interdigital electrodes. (D) Remove the limiting parts and obtain the sensing module.

### 4.3 Characterization and measurements

#### 4.3.1 Characterization and measurements of the NTAS

A programmable electrometer (Keithley, model 6514) was adopted to test the *V*_OC_. The data was transmitted to a computer through an acquisition card (National Instruments, USB‐6346) and processed by a software, LabVIEW.

1. Rewarm the citrated porcine blood sample to 37°C;
2. Replace the used probe and cuvette with new ones and open the thermostatic control module to preheat the cuvette at 37°C;
3. Pipet 20 μL CaCl_2_ solution to the cuvette (providing the necessary calcium ions for blood coagulation);
4. Pipet 1 mL blood sample to an activation tube, turn the activation tube upside down for five times, and let it stand for 1 min;
5. Pipet 340 μL activated blood sample to the cuvette;
6. Lift the cuvette and run the rotating module;
7. Start collecting data and lasts for about 30 min;
8. Save data and obtain the trace and parameters (R, K, Angle, and MA).

## AUTHOR CONTRIBUTIONS

Baocheng Wang designed the study, performed experimental measurements, designed the detection and analysis program, and wrote the manuscript. Xuelian Wei contributed to the fabrication of the NTAS, the data analysis, and the schematic of the article. Hanlin Zhou contributed to the mechanical design of the NTAS‐TEG. Enyang Zhang and Xiaole Cao contributed to the tests of blood samples. Zhong Lin Wang and Zhiyi Wu conceived the research and supervised all aspects of the work.

## CONFLICT OF INTEREST STATEMENT

The authors declare no conflicts of statement.

## Supporting information

Supporting Information

Supporting Information

Supporting Information

Supporting Information

Supporting Information

## Data Availability

The data that support the findings of this study are available from the corresponding author upon reasonable request.
