# Supplementary material for: Viscoelastic blood coagulation testing system enabled by a non‐contact triboelectric angle sensor
Source: Exploration (Beijing). 2023 Nov 23;4(1):20230073. doi: 10.1002/EXP.20230073 (PMC10867393; doi:10.1002/EXP.20230073)
Supplement: Supplementary file 1 — Supporting Information [file EXP2-4-20230073-s001.docx]

**Viscoelastic blood coagulation testing system enabled by a non-contact triboelectric angle sensor**

**Authors:**

Baocheng Wang^1,2 †^, Xuelian Wei^1,2 †^, Hanlin Zhou^1^, Xiaole, Cao^1,2^, Enyang Zhang^1^, Zhong Lin Wang^1,3^*, Zhiyi Wu^1,2^*

**Affiliations:**

^1^Beijing Institute of Nanoenergy and Nanosystems, Chinese Academy of Sciences, Beijing 101400, China.

^2^School of Nanoscience and Technology, University of Chinese Academy of Sciences, Beijing 100049, China.

^3^Georgia Institute of Technology, Atlanta, GA 30332, USA.

^†^ These authors contributed equally to this work.

* E-mail: wuzhiyi@binn.cas.cn (Z. Wu); zhong.wang@mse.gatech.edu (Z. L. Wang)

**Supporting information includes:**

**Figure S1. Photographs of the components of the NTAS-enabled TEG**

**Figure S2. Specific dimensions of the sensing module.**

**Figure S3. Fabrication process of the sensing module.**

**Figure S4. Simulated electric potential diagram of the NTAS array.**

**Figure S5. Output performance of the NTAS.**

**Figure S6. Photograph of the viscoelastic blood coagulation detection and analysis system.**

**Table S1. Rotating process of the rotating platform every cycle.**

**Table S2. Simulation parameters of static field simulation by Ansys Workbench.**

**Table S3. Simulation parameters of the electrostatic field simulation by COMSOL Multiphysics.**

**Table S4. Coagulation parameter value of hypercoagulation, normal, and hypocoagulation quality control samples.**

**Supporting Movie 1: Preparation of a test by the NTAS-enabled TEG**

**Supporting Movie 2: Motion details and corresponding voltage waveforms of the NTAS during tesing a blood sample**

**Supporting Movie 3: Test of a hypocoagulable quality control sample by the NTAS-enabled TEG.**

**Supporting Movie 4: Test of a hypercoagulable quality control sample by the NTAS-enabled TEG**

^
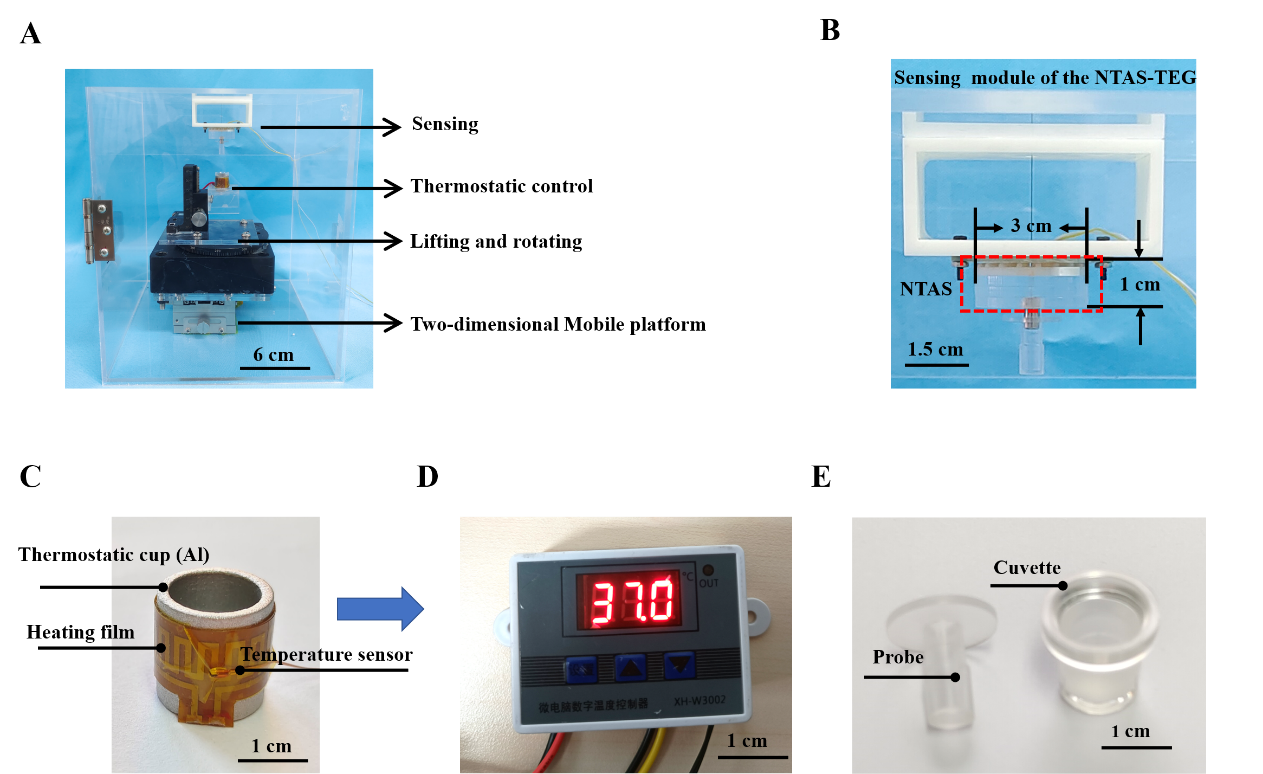
^

**Figure S1. Photographs of the components of the NTAS-enabled TEG.** A) Photograph of the NTAS-TEG. B) Photograph of the NTAS-based sensing module. C) Photograph of the thermostatic cup covered with a heating film and a temperature sensor D) Photograph of the temperature control unit. E) Photographs of the probe and the cuvette.

^
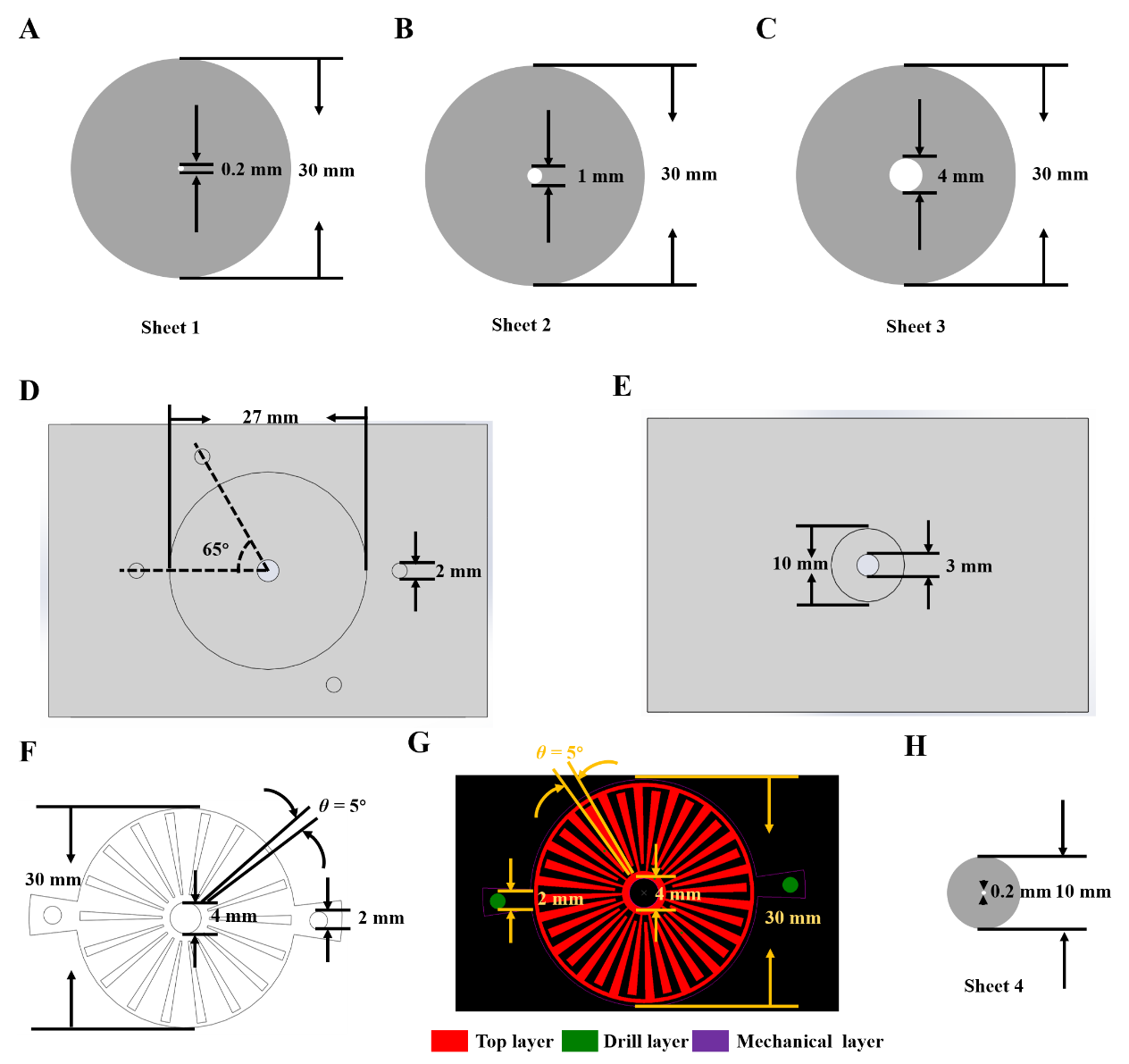
^

**Figure S2. Specific dimensions of the sensing module.** A)-C) Dimensions of rotor’s acrylic bases. D) Dimensions of the box bottom view. E) Dimensions of the box top view. F) Dimensions of the functional layer of the rotor. G) Dimensions of the interdigital electrodes board. H) Dimensions of the acrylic base on which the top end torsion wire is fixed.

^
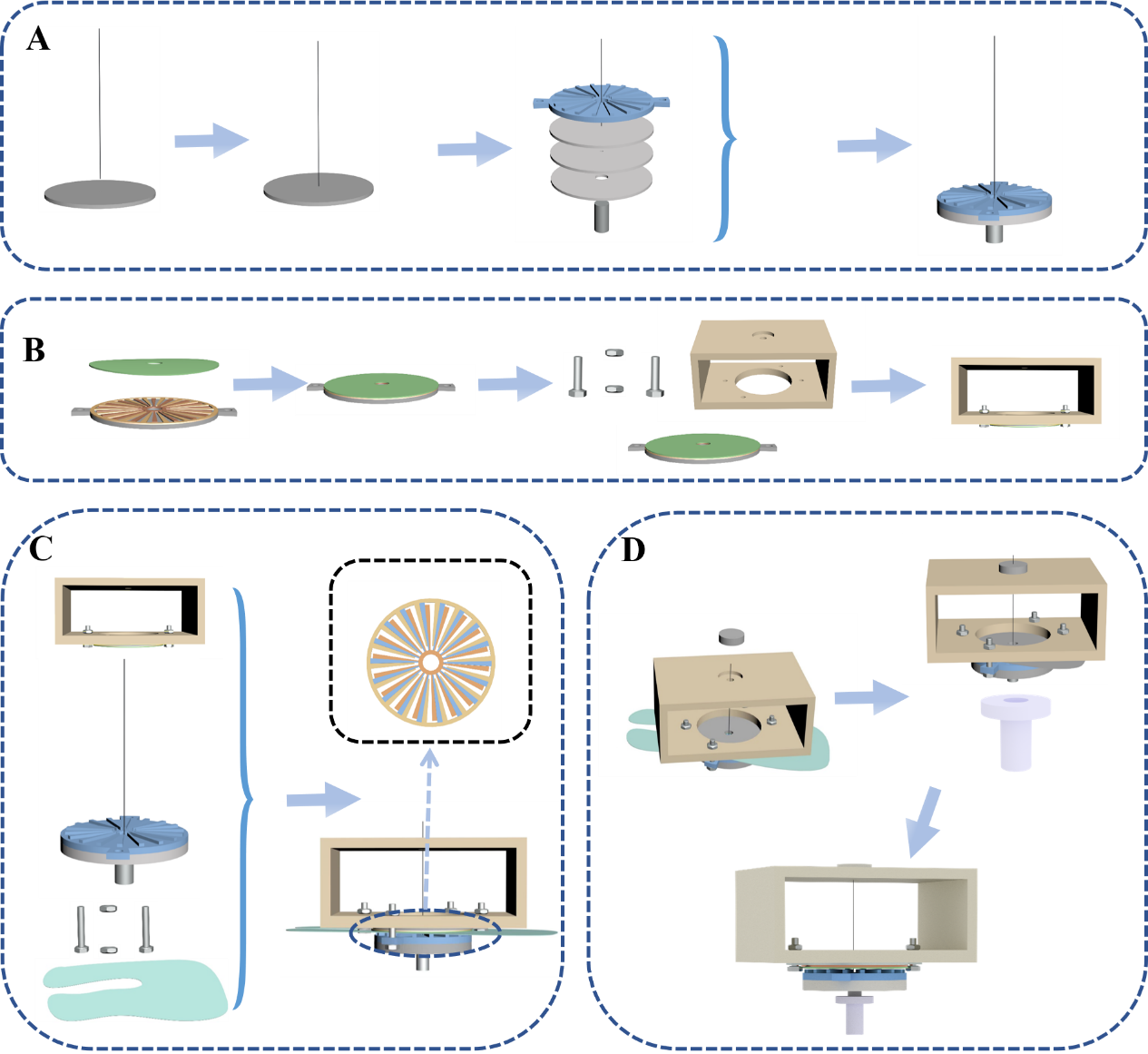
^

**Figure S3. Fabrication process of the sensing module.** A) Fabrication of the NTAS’s rotor integrated with a torsion wire and a shaft. B) Fabrication of the NTAS’s stator. C) Set the relative position of the rotor and the stator. The relative position of the rotor grids and stator grids are shown in the inset. D) Remove the limiting parts and obtain the sensing module.

^
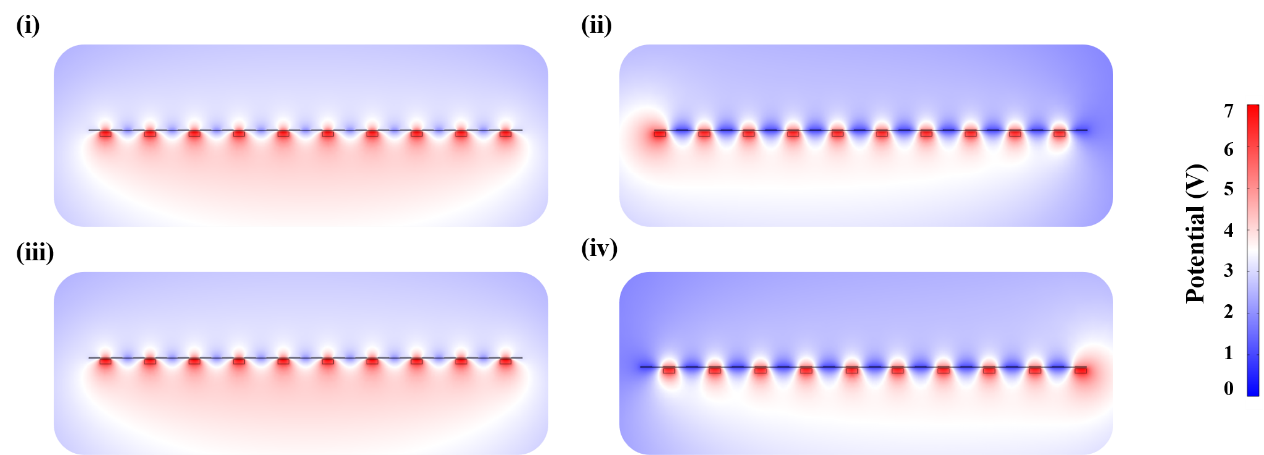
^

**Figure S4. Simulated electric potential diagram of the NTAS array.**

^
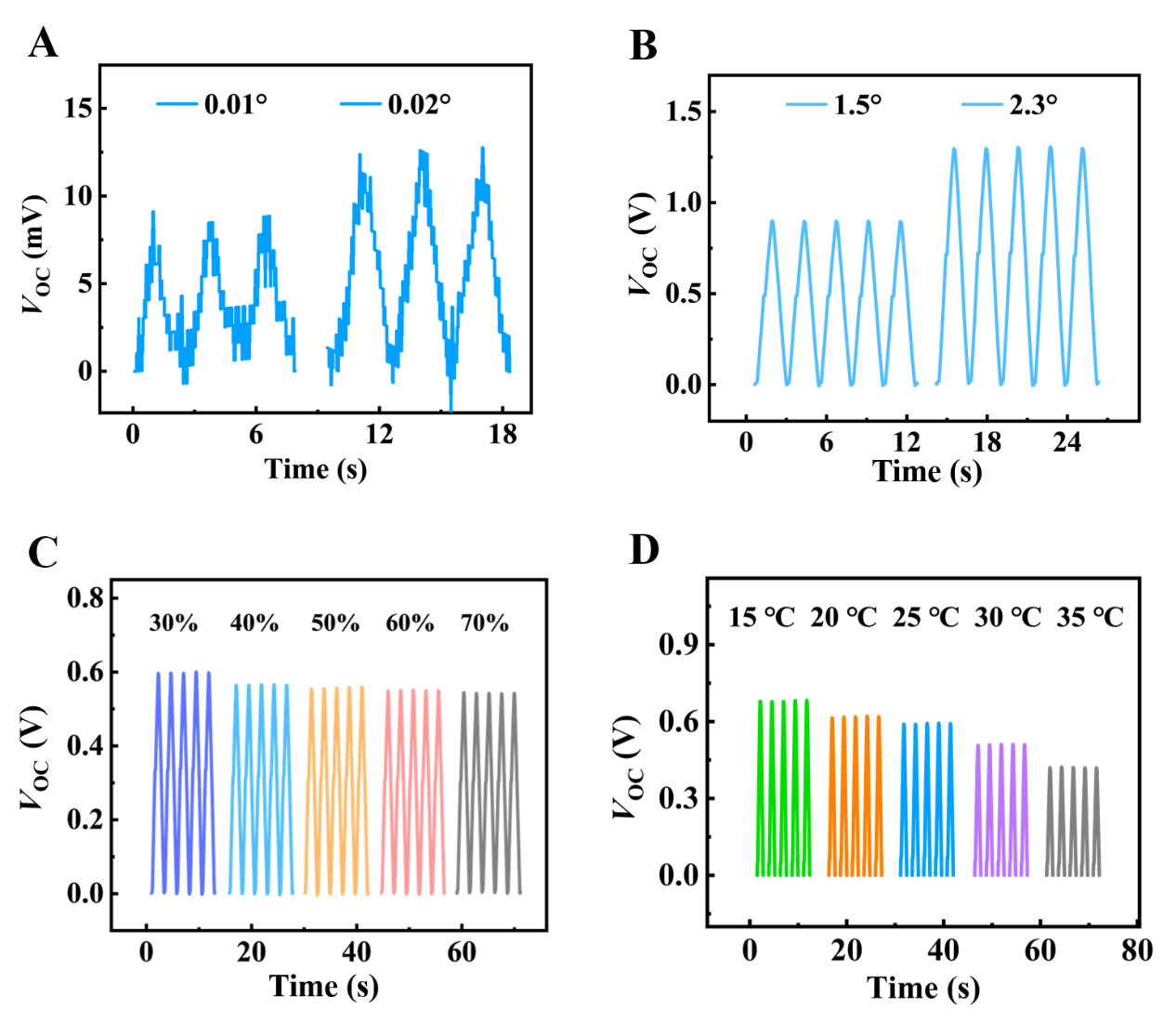
^

**Figure S5. Output performance of the NTAS.** A) *V*_OC_ of 0.01° and 0.02°. B) *V*_OC_ of 1.5° and 2.3°. C) *V*_OC_ of humidity from 30% to 70% (1°, 25 ℃). D) *V*_OC_ of temperature from 15 ℃ to 35 ℃ (1°, 30%).

^
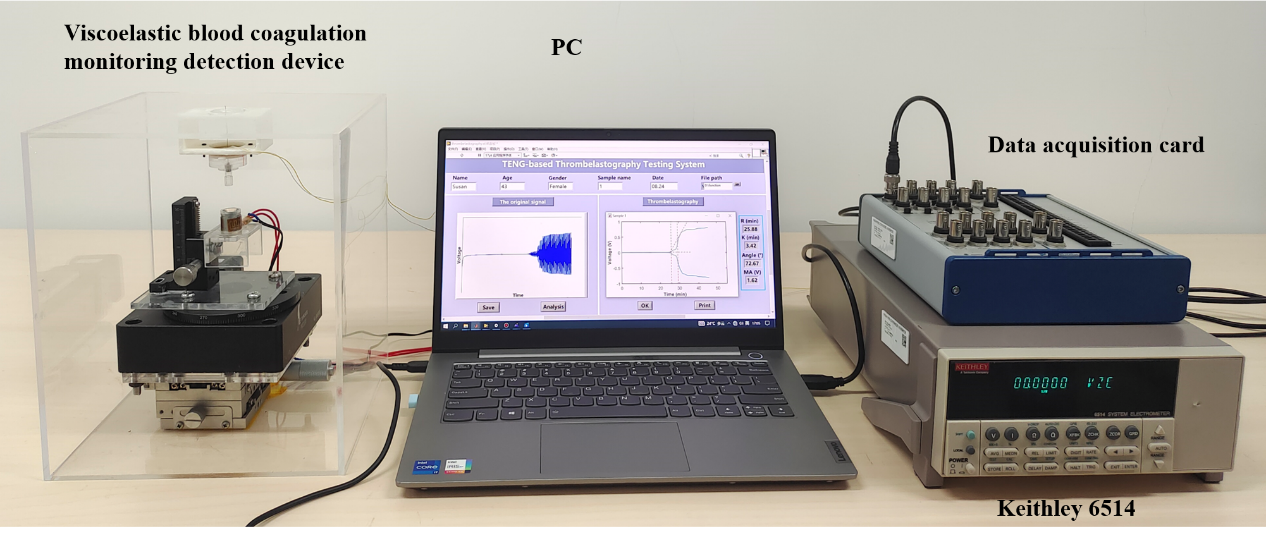
^

**Figure S6. Photograph of the viscoelastic blood coagulation detection and analysis system.**

**Table S1. Rotating process of the rotating platform every cycle.**

| Initial position: 0°; Speed: 0.95° s^-1^ | |
| --- | --- |
| 1 | Loop starts |
| 2 | -2.375° |
| 3 | 4.75° |
| 4 | -2.375° |
| 5 | Loop ends |

**Table S2. Simulation parameters of static field simulation by Ansys Workbench.**

| Materials | Density (Kg m^-3^) | Shear modulus (GPa) | Poisson’s ratio |
| --- | --- | --- | --- |
| Titanium alloy | 4429 | 41.5 | 0.34 |
| Steel | 7850 | 76.9 | 0.3 |
| Plastic (cuvette, probe) | 1392 | 1.0 | 0.4 |
| Blood | 1000 | 4 × 10^-7^ | 0.4 |

**Table S3. Simulation parameters of the electrostatic field simulation by COMSOL Multiphysics.**

| 1 mm (width of E_1_ of NTAS’s simplified simulation model) corresponds to 5° (central angle of actual NTAS’s electrode grids) when simulating the rotation angle sensing characteristics of the NTAS. | |
| --- | --- |
| Width of E_1_,E_2_ | 1 mm |
| Width of photocurable resin grids | 1 mm |
| Thickness of E_1_,E_2_ | 35 μm |
| Thickness of photocurable resin grids | 400 μm |
| Thickness of PTFE | 50 μm |
| Dielectric coefficient | 2 |
| Surface charge density of PTFE | -6 × 10^-8^ C m^-2^ |
| Gap between electrodes and the dielectric layer | 5 μm |
| Gap between PTFE and photocurable resin | 100 μm |

**Table S4. Coagulation parameter value of hypercoagulation, normal, and hypocoagulation quality control samples.**

|  | Hypercoagulation quality control sample | Normal quality control sample | Hypocoagulation quality control sample |
| --- | --- | --- | --- |
| R (min) | 2.5 | 6.06 | 25.7 |
| K (min) | 1.9 | 2.85 | 3.4 |
| Angle (°) | 76.7 | 67.8 | 65.8 |
| MA (V) | 2.0 | 1.88 | 1.6 |
